# Probabilistic tractography in the ventrolateral thalamic nucleus: cerebellar and pallidal connections

**DOI:** 10.1007/s00429-020-02076-9

**Published:** 2020-05-03

**Authors:** Esther A. Pelzer, K. Amande M. Pauls, Nina Braun, Marc Tittgemeyer, Lars Timmermann

**Affiliations:** 1 Department of Neurology, University Hospital Cologne, Cologne, Germany; 2 Max-Planck Institute for Metabolism Research, Gleulerstr. 50, 50931 Cologne, Germany; 3 Department of Neurology, Helsinki University Central Hospital, Helsinki, Finland; 4 Department of Clinical Neurosciences (Neurology), University of Helsinki, Helsinki, Finland; 5 BioMag Laboratory, HUS Medical Imaging Center, University of Helsinki and Helsinki University Hospital, Helsinki, Finland; 6 Department of Neurology, University Hospital Marburg, Marburg, Germany

**Keywords:** Ventrolateral thalamus, Basal ganglia, Cerebellum, Probabilistic tractography, Qualitative and quantitative analyses

## Abstract

The ventrolateral thalamic nucleus (VL), as part of the ‘motor thalamus’, is main relay station of cerebellar and pallidal projections. It comprises anterior (VLa) and posterior (VLpd and VLpv) subnuclei. Though the fibre architecture of cerebellar and pallidal projections to of the VL nucleus has already been focus in a numerous amount of in vitro studies mainly in animals, probabilistic tractography now offers the possibility of an in vivo comparison in healthy humans. In this study we performed a (a) qualitative and (b) quantitative examination of VL-cerebellar and VL-pallidal pathways and compared the probability distributions between both projection fields in the VL after an (I) atlas-based and (II) manual-based segmentation procedure. Both procedures led to high congruent results of cerebellar and pallidal connectivity distributions: the maximum of pallidal projections was located in anterior and medial parts of the VL nucleus, whereas cerebellar connectivity was more located in lateral and posterior parts. The median connectivity for cerebellar connections in both approaches (manual and atlas-based segmentation) was *VLa* > *VLpv* > *VLpd*, whereas the pallidal median connectivity was *VLa* ~ *VLpv* > *VLpd* in the atlas-based approach and *VLpv* > *VLa* > *VLpd* in the manual approach.

**Electronic supplementary material:**

The online version of this article (10.1007/s00429-020-02076-9) contains supplementary material, which is available to authorized users.

## Introduction

The ventrolateral (VL) thalamic nucleus is main relay station of cerebellar and basal ganglia (BG) projections to the cortex (Asanuma et al. 1983; Sidibe et al. 1997).

Both, the deep cerebellar nuclei (esp. dentate nucleus and interposed nuclei) and output nuclei of the basal ganglia (substantia nigra, reticulate part, and the globus pallidus, internal part) project to the thalamus with a considerable amount of overlap, as shown in the monkey and human species (Pelzer et al. 2016; Sakai et al. 1996). In this study we specifically analysed the qualitative and quantitative fibre distribution of pallidal and cerebellar connections in a (i) manual- and (ii) atlas-based segmentation of VL nucleus. By the use of probabilistic tractography we present a detailed in vivo description and a quantitative comparison between these fibre projections.

## Results

12 healthy right-handed (> 7th percentile) native German speakers (10 women, 2 men) with a mean age of 25 (± 4.4) years, without history or signs of neurological disease, were included in the MR analysis. Informed consent was obtained from all individual participants included in the study. For overview of methods please see Supplementary Material.

**Fig. 1:**
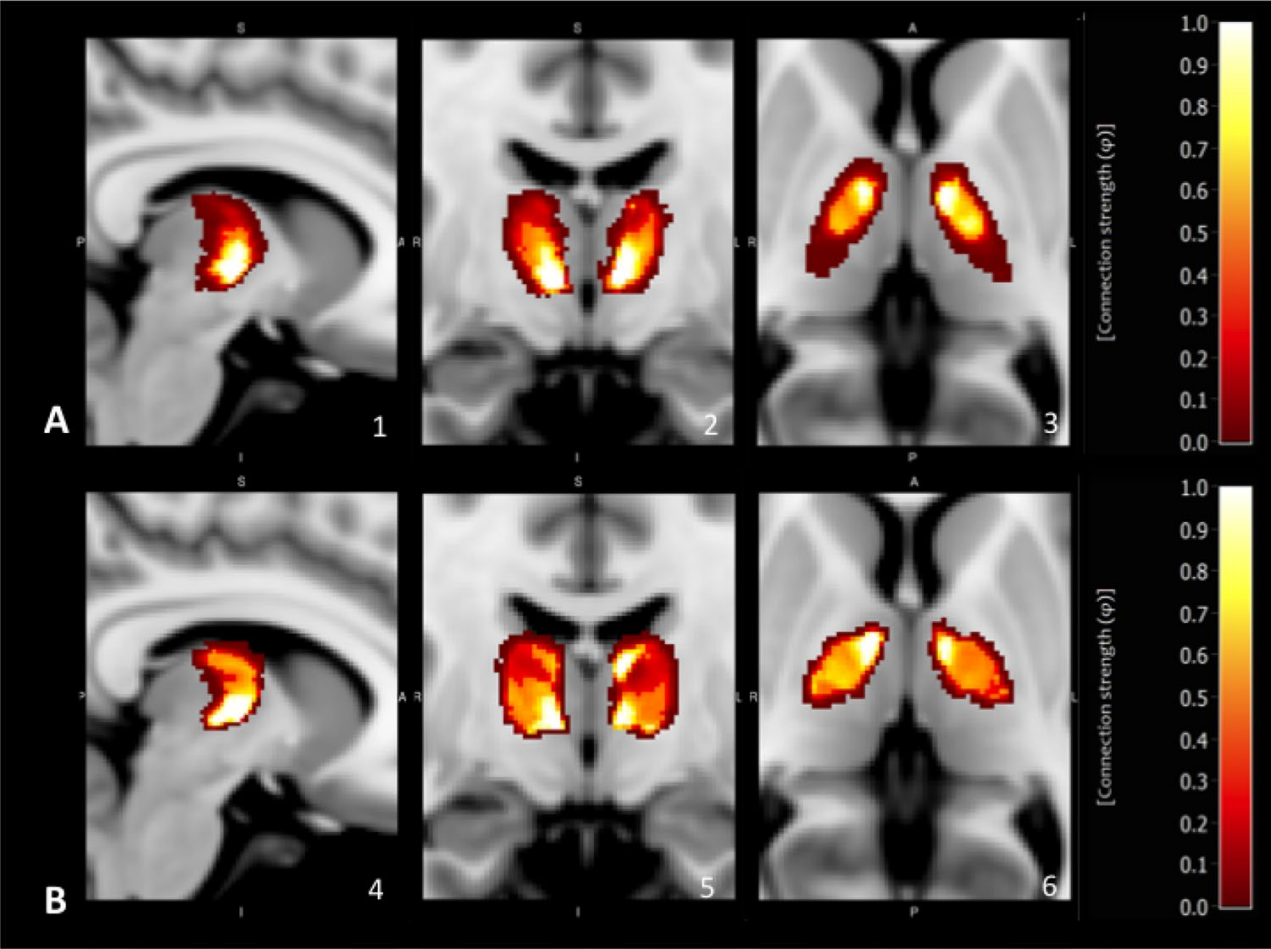
Qualitative analysis of VL-pallidal connection strength: **a** Manual segmentation **b** Atlas-based segmentation. All results are depicted in the *x*, *y* and *z* plane; (1, 4) *x* = 81, (2, 5) *y* = 116; (3, 6) *z* = 76. Connection strength *φ*: 0 = no connectivity (red); 1 = maximum connectivity (yellow)

**Fig. 2:**
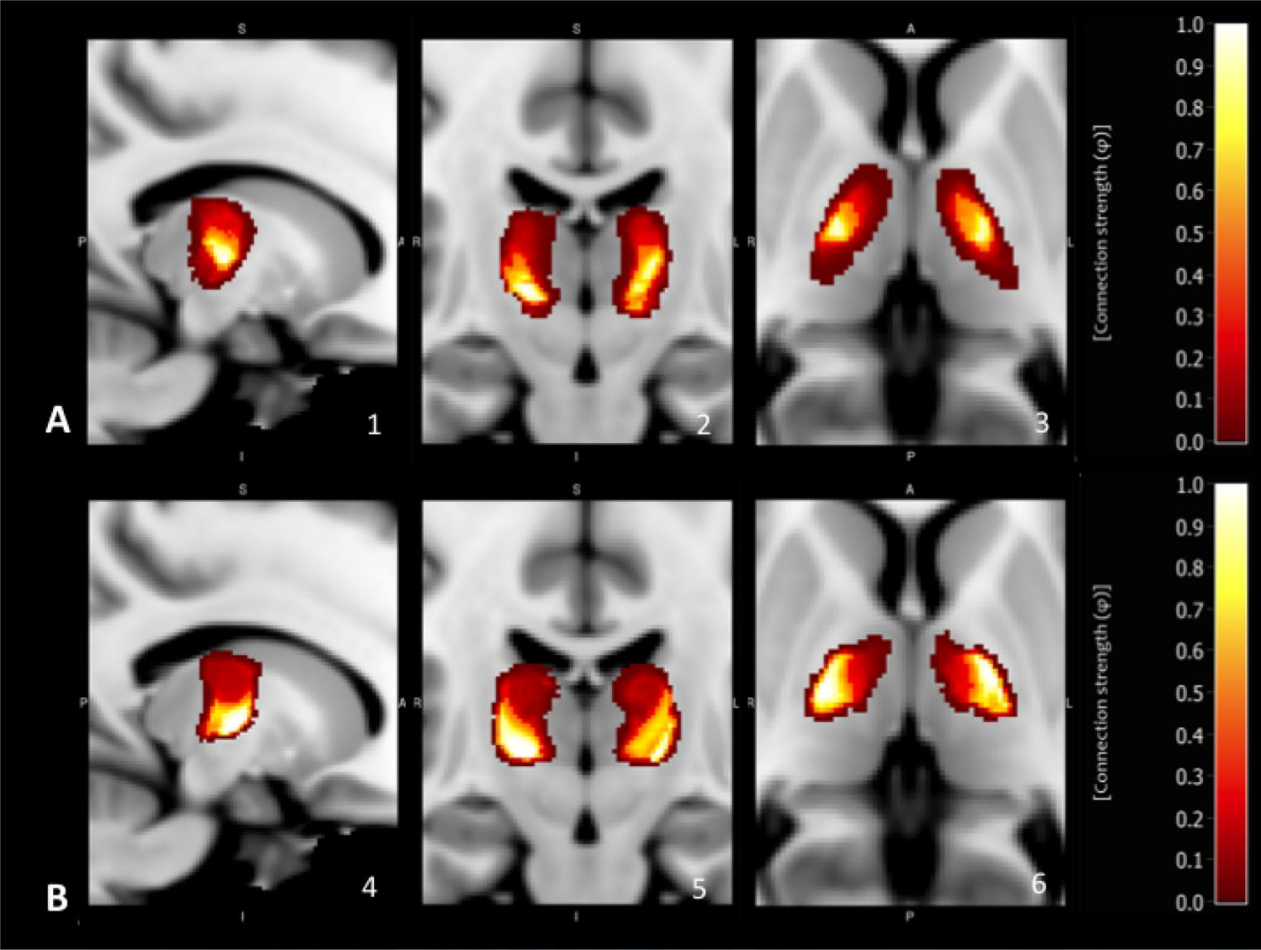
Qualitative analysis of VL-cerebellar connection strength: **a** Manual segmentation, **b** Atlas-based segmentation. All results are depicted in the *x*, *y* and *z* plane; (1, 4) *x* = 81, (2, 5) *y* = 116; (3, 6) *z* = 76. Connection strength *φ*: 0 = no connectivity (red); 1 = maximum connectivity (yellow)

**Fig. 3:**
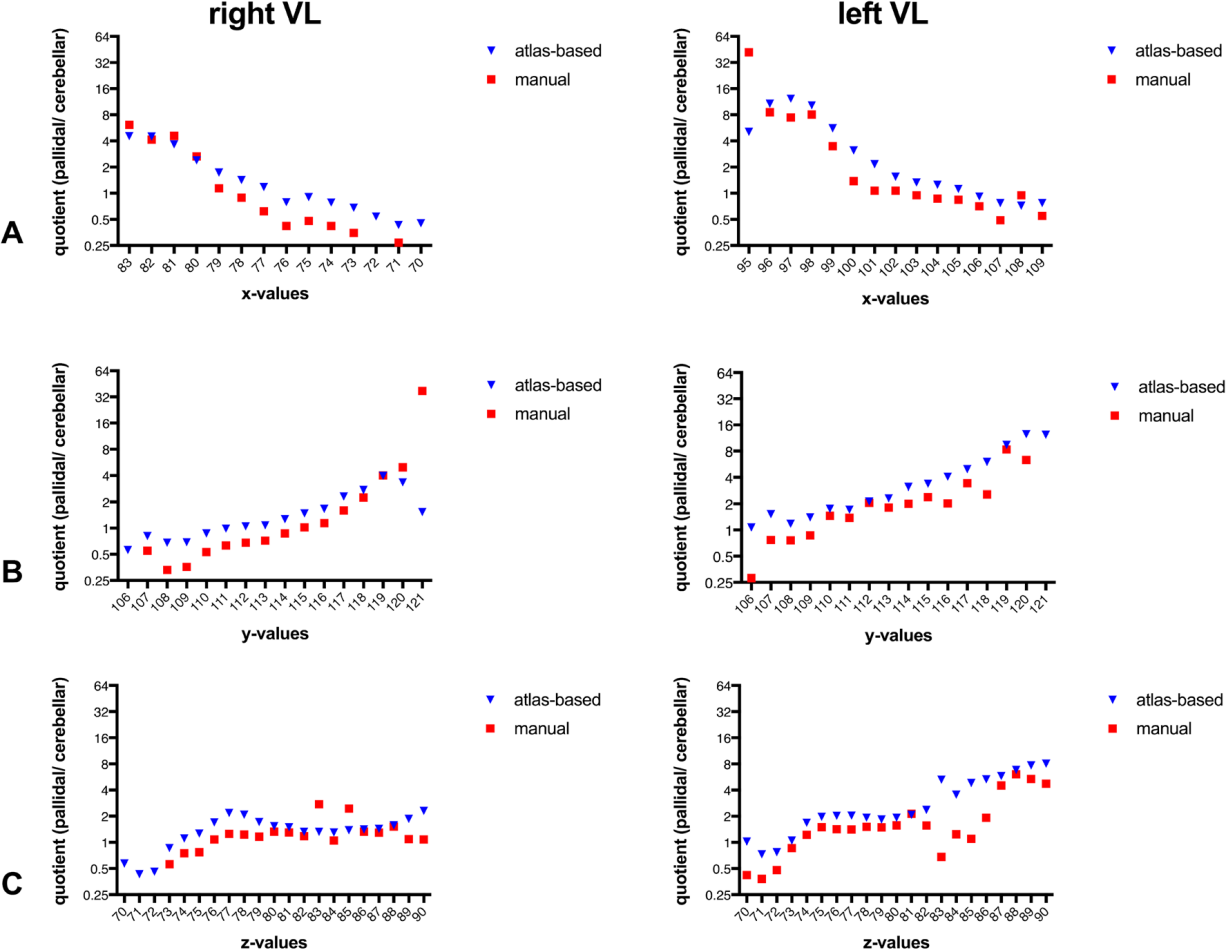
Quantitative analysis of VL-connectivity. Presented is the quotient derived from pallidal connectivity values divided by cerebellar values for the right and left hemisphere in the **a***x*-plane, **b***y*-plane and **c***z*-plane

Interestingly, atlas-based segmentation procedure revealed a 41% (SD 2.70) bigger VL masks on the left side and 42% (SD 2.28) bigger VL masks on the right side than the manual outlining procedure; differences were mainly located in top and bottom border regions. In the VL both, manual and atlas-based segmentation, yielded to highly congruent specific patterns in the qualitative analysis of fibre architecture: (1) pallidal connectivity had a maximum in more anterior and medial parts of the VL, whereas cerebellar connectivity was more located in lateral and posterior parts (see Figs. 1 and 2). In a second step we quantified thalamic connectivity for both, the manual and atlas-based method: Both methods showed a considerable amount of overlap between cerebellar and pallidal projections (see Fig. 3), with a medial-to lateral decreasing quotient of pallidal compared to cerebellar connectivity (see Fig. 3a), an inferior-to-superior (see Fig. 3c) increase of pallidal connectivity compared to cerebellar connectivity and a posterior to anterior increase of pallidal compared to cerebellar connectivity (see Fig. 3b). As one example of descriptive statistics we determined the median connectivity values in both, manual and atlas-based segmentation of the VL. For cerebellar connections, both approaches revealed a similar connectivity pattern (*VLa* > *VLpv* > *VLpd)*, whereas the pallidal median connectivity values differed between the atlas-based (*VLa* ~ *VLpv* > *VLpd)* and the manual *(VLpv* > *VLa* > *VLpd)* approach.

## Discussion

(1) We found high connectivity values to the VLa and VLp regions for both projection systems, with an anterior-posteriorly increasing gradient for cerebellar projections and a posterior-anteriorly increasing gradient for cerebellar projections. Our results fit well with the current anatomical knowledge [see Fig. 6 in Sakai et al. (1996)]. The pallido-thalamic territory includes VApc (not part of our investigation), VLa and dorsal part of VLp, and occasional patches of pallidal label in VLpv and the anteromedial part of VLp. The density of pallido-thalamic projections decreases along an anterior to posterior gradient. Conversely, the density of cerebello-thalamic projections increases along the same gradient, with the cerebello-thalamic territory extending anteriorly beyond the cell-sparse zones of the ventral part of VLp, anteromedial part of VLp, dorsal part of VLp to include VLa and VApc also (Sakai et al. 1996). We found large differences in the resulting amount of voxels, which were included in the segmentation procedure between the atlas-based and manual segmentation; still the (1) qualitative and (2) quantitative visualization remained stable. However the descriptive statistic, as here shown for the median connectivity values per subnuclei, evoked remarkable differences between both segmentation procedures; this yields to the conclusion that next to hard segmentation procedures, a common feature in probabilistic tractography, also the examination of the individual connectivity values seems absolutely necessary to depict the whole truth of fiber distribution via probabilistic tractography (Jbabdi et al. 2015).

The ventrolateral thalamus is one of the main target regions in stereotactic treatments for movement disorders like essential tremor (Vaillancourt et al. 2003) or Parkinsonian tremor (Benabid et al. 1996).

For neurosurgeons, problems in optimal targeting already begin in the diverse nomenclatures of the ventrolateral thalamus, for overview please see Krack et al. (2002). The most commonly used nomenclatures in humans are e.g. the ones proposed by Hassler (1982) and Hirai et al. (1989). The most commonly used nomenclatures in primates are e.g. the ones proposed by Ilinsky and Kultas-Ilinsky (2002), Olszewski (1952) and Macchi and Jones (1997). These diverse nomenclatures make the interpretation of cerebellar and basal ganglia fibre distributions and optimal targeting of stereotactic surgery in the ventrolateral thalamus challenging.

Beside, also the distribution and the “communication” between the cerebellar and pallidal projection system in the ventrolateral thalamus are discussable. Whereas some researchers claim a strong segregation of cerebellar and basal ganglia projections, we recently found hints for an informational exchange between these two systems [for detailed discussion please see Hintzen et al. (2018)].

Another fact is, that the thalamic target for stereotactic surgery in the treatment of e.g. tremor is not readily visible on conventional magnetic resonance imaging. Knowledge is based on anatomy by diverse animals and methods (e.g. immunhistochemistry or myelin staining) or post-mortem analyses [for detailed overview please see Hintzen et al. (2018)]. Diffusion MRI and tractography nowadays offers the opportunity to depict anatomical connections in vivo in the human species [e.g. Jbabdi et al. (2015); Lerch et al. (2017)]. But we claim to be cautious in the application of hard segmentation procedures without including knowledge of anatomical in vitro animal studies and postmortem studies regarding the fibre distribution in the ventrolateral thalamus. The exact targeting of cerebellar termination fields in stereotactic surgery is though of utmost importance: For example regarding essential tremor, tremor-related activity is most prominent in cerebellar recipient subdivisions of the ventrolateral thalamus, that is in Jones’ nomenclature, the VLp [for a review see Hamani et al. (2006)]. A functional micro-electrode mapping of ventral thalamus in essential tremor, for example, showed that the inferior posterior ventrolateral thalamus and its border region plays a key role in essential tremor pathophysiology (Pedrosa et al. 2018); stereotactic lesioning in exactly this localisation may relieve symptoms and will reduce the cause of relevant side effects most effectively. Additionally the higher variability in the z-values in the manual based segmentation in Fig. 3 shows, that due to the less contrast and the higher difficulty in finding the boundaries of the ventrolataral thalamus a higher variability in connectivity values derived. A fact that also has high implications for the planning of stereotactic coordinates in neurosurgery in the ventrolateral thalamus because reducing effectiveness and increasing side effects.

## Electronic supplementary material

Below is the link to the electronic supplementary material.

Supplementary file1 (DOCX 170 kb)
