# Supplementary material for: Probabilistic tractography in the ventrolateral thalamic nucleus: cerebellar and pallidal connections
Source: Brain Struct Funct. 2020 May 3;225(5):1685–9. doi: 10.1007/s00429-020-02076-9 (PMC7286851; doi:10.1007/s00429-020-02076-9)
Supplement: Supplementary file 1 — Supplementary file1 (DOCX 170 kb) [file 429_2020_2076_MOESM1_ESM.docx]

**Methods**

*MR- data acquisition*

Diffusion-weighted MRI (dMRI) and high-resolution three-dimensional (3D) T1- and T2-weighted images were acquired on a Siemens 3T Tim Trio scanner.

The acquisition parameters of the anatomical data sets were for the T1 image: MPRAGE; TR=1930 ms, TI=650 ms, TE=5.8 ms, resolution 1.0 x 1.0 x 1.25 mm, flip angle 18°, sagittal and for the T2 image: RARE; TR=3200 ms, TE=458 ms, 176 sagittal slices, resolution 1.0 x 1.0 x 1.0 mm^3^.

The diffusion-weighted data (dMRI) sets were acquired using echo planar imaging (EPI; TR=12000 ms, TE=100 ms, resolution 1.7x1.7x1.7 mm^3^, flip angle: 90°, Field of View: 220x220x122 mm^3^, bandwidth: 1345 Hz/pixel, orientation: axial, data matrix: 128x128x72, PAT factor 2, partial Fourier 6/8, no cardiac gating) with double-spin echo preparation (Reese et al., 2003).

Diffusion weighting was isotropically distributed along 60 diffusion-weighted directions (b-value=1000 s/mm^2^) and is called high angular resolution diffusion imaging (HARDI) technique (Berman et al., 2013; Mori and Tournier, 2014). HARDI is capable of discriminating multiple fiber populations crossing within the same voxel. The higher angular resolution provides a more accurate representation of the 3D pattern of water diffusion within a voxel; herewith HARDI can follow white matter tracts through regions of crossing fibres, like necessary for the decussation of the superior cerebellar peduncle. Fiber tracking with HARDI can follow white matter tracts through regions of crossing fibers whereas DTI fiber tracking would terminate or provide erroneous results in these same areas (Behrens et al., 2007; Berman et al., 2013; Campbell et al., 2005; Perrin et al., 2005).

Additionally, in each subject seven data sets without diffusion weighting were acquired initially and interleaved after each block of 10 diffusion-weighted images as anatomical reference for motion correction. To increase signal-to-noise ratio, dMRI scanning was repeated three times for averaging. The dMRI data were acquired after the T1- and T2-weighted images in the same scanner reference system.

*Pre-processing procedure*

All processing steps were performed using FSL (version 4.1.9; http://fsl.fmrib.ox.ac.uk/fsl). Motion and eddy current correction of diffusion‐weighted images was applied to all images using six degrees of freedom applying a rigid-body registration. All baseline b0 images were aligned to a reference b0 image and the resulting linear transformation matrices were then applied to the diffusion‐weighted images following each baseline b0 image. Scanning was repeated three times and this information was included for modelling of diffusion data to improve signal to noise ratio for every direction.

- For pre-processing of anatomical images, 3D-T1-weighted images were reoriented to the sagittal plane through the anterior and posterior commissures. This 3D-T1-weighted image served as individual structural space for each subject and was used as high-resolution image for further analysis.

After reorientation, the 3D-T1 image and a 3D-T2-weighted image were linearly co-registered via 12 degrees of freedom (DOF) transformation using FLIRT (Jenkinson and Smith, 2001); the registration procedure was checked visually for every subject. For tractography, linear matrices for registration of the diffusion-weighted data to structural space were calculated with 12 degrees of freedom and inverted. The inverted matrices were used for the transformation of region of interest (ROI) into diffusion space to run probabilistic tractography.

*Post-processing procedure and outlining of ROI masks*

All data sets were first controlled for completeness, especially in dentate regions, magnitude of artefacts, sufficient SNR and homogeneity. Masks were drawn on individual registered T1-/T2-weighted images using *fslview* (<http://fsl.fmrib.ox.ac.uk/fsl/fslview>).

The following segmentation protocol was chosen for (I) *manual outlining* of VL region, which was defined as the seed region: anatomically the VL region (including VLa, VLp) was outlined using individual T1 images and an overlaying 3T based diffusion tensor eigenvector map (Wiegell et al., 2003). Segmentation was carried out using axial slices starting with the slice with the largest dimensions of the VL region, and was then continued in consecutive slices in dorsal and ventral direction. The lateral (internal capsule) and medial (intralaminar nuclei, medullary laminary) boundaries of the VL region were properly distinguishable from T1 images. The posterior border of VL (adjacent to the pulvinar) was easily discernible on T1 images. To confirm the dorsal borders and the anterior border, the diffusion tensor eigenvector map and comparison with available atlases of the human brain [Schaltenbrand and Wahren (1977); Mai et al. (1997); Morel (2007); Krauth et al. (2010)] were used. Masks were outlined by one initial observer were then controlled for consistency on axial and coronal sections by a second observer. Both observers were trained specialists in thalamic anatomy by Prof. Jürgen K. Mai, University of Dusseldorf. We chose the manual outlining based on individual anatomy (shape of the thalamus, position of the internal capsule and the size of the size of the ventricle) because registration algorithms work moderately in regions close to the ventricle. The DN, as the main projection zone of the deep cerebellar nuclei, and the GP were chosen as target points [for more details of the segmentation protocol for the DN and the GP see Pelzer et al. (2013)

For (II) *atlas based segmentation* VLa, VLpd and VLpv subnuclei were concatenated to the left and right VL based on the anatomical information of the Krauth atlas (Krauth et al., 2010). We chose the digitalized version of the atlas of the human thalamus of Morel et. al (Jakab et al., 2012; Krauth et al., 2010; Morel, 2007) with a high resolution in axial (0.9 mm), sagittal (0.9 mm) and coronar (1.8 mm) direction and a subnuclei differentiation based on following neurochemical markers (parvalbumin, calbindin D 28-K and calretinin). Due to its availability in the MNI-1mm standard space (Jakab et al., 2012; Krauth et al., 2010) the detailed examination of thalamic fibre architecture is nowadays possible.

For an overview of resulting masks of manual and atlas-based segmentation please see Fig. 1.

*Diffusion tractography and Visualisation*

Fiber tracking based on this HARDI technique can follow white matter tracts through regions of crossing fibers, as we have already proofed for our data in a previous paper specifically focussing on the anatomical fibre course in regions of the decussation of the of the superior cerebellar peduncle (Pelzer et al., 2013).

The FDT-toolbox of FSL-Software was applied for probabilistic tractography (<https://fsl.fmrib.ox.ac.uk/fsl/fslwiki/FDT/UserGuide>). The affine transformation matrices generated during registration procedure were implemented into PROBTRACKX to transform seed- and target masks from structural space into the diffusion space, because all tractography was done in diffusion space.

We calculated connectivity distributions between seed- and target regions (samples n=5000, steps n=2000, step length 0.5 mm, curvature threshold 0.2, corresponding to a minimum angle of approximately ±80 degrees). We added a path distribution function to correct for differences in distance between seed and target regions.

Results were checked for anatomical reliability, especially in the regions of the decussation of the superior cerebellar peduncle. Anatomical NOT-regions were not excluded, because the targets of the VL region (pallidum, dentate nucleus) were included in one tractographic analysis simultaneously in order to get the typical “*seed_to_target” masks* (for more details please see: **‘Connectivity-based seed classification’;** <https://fsl.fmrib.ox.ac.uk/fsl/fslwiki/FDT/UserGuide>**).** Because of inability of diffusion tractography to differentiate between afferent and efferent connections, labelling was assigned based on prior knowledge of histologically established fibre projections (cerebello-VL, pallido-VL).

Despite correcting for the distance of the paths by the function “path distribution”, absolute pallidal connectivity values were “over-representative” in the VL region compared to cerebellar connectivity values; we were therefore not able to run the **‘connectivity-based seed classification’ (specifically the ‚find_the_biggest’ algorithm).**

Therefore resulting cerebello-VL and pallido-VL connectivity maps were normalised by the geometric mean of the robust maxima of every individual connectivity map.

After transformation into MNI152 1-mm standard space, the arithmetic mean of all normalised maps was determined for visualization. Results were rescaled from 0-1 to render the connectivity maps of cerebellar and pallidal projections with the VL region visually comparable.

In order to avoid additional interpolation artefacts caused by registering the small thalamic nuclei, we did not transfer the VL nuclei of the Morel atlas (which are provided by Krauth et al. (2010) in MNI-1 mm standard space) to the native subject space, but transferred the cerebello-VL and pallido-VL connectivity maps non-linearily to the MNI152-1mm standard space for further post-processing and display.

After calculation of the connectivity map (to the pallidum and to the cerebellum), the probability values for individual voxels of the VL map were also used to calculate the arithmetic mean connectivity for the thalamic subnuclei VLa, VLpd and VLpv by segmenting the resulting individual probability maps based on the histologically identified thalamic nuclei provided by the Morel atlas (Jakab et al., 2012; Krauth et al., 2010; Morel, 2007). Subnuclei were ranked based on mean pallidal and dentate connectivity per subnucleus.

**References**

Behrens, T.E.J., et al., 2007. Probabilistic diffusion tractography with multiple fibre orientations: What can we gain? Neuroimage. 34**,** 144-155.

Berman, J.I., et al., 2013. High angular resolution diffusion imaging probabilistic tractography of the auditory radiation. AJNR. American journal of neuroradiology. 34**,** 1573-1578.

Campbell, J.S.W., et al., 2005. Flow-based fiber tracking with diffusion tensor and q-ball data: validation and comparison to principal diffusion direction techniques. NeuroImage VL - IS - SP - EP -. 27**,** 725-736.

Jakab, A., et al., 2012. Generation of individualized thalamus target maps by using statistical shape models and thalamocortical tractography. AJNR. American journal of neuroradiology. 33**,** 2110-2116.

Jenkinson, M., Smith, S., 2001. A global optimisation method for robust affine registration of brain images. Medical Image Analysis. 5**,** 143-156.

Krauth, A., et al., 2010. A mean three-dimensional atlas of the human thalamus: generation from multiple histological data. Neuroimage. 49**,** 2053-2062.

Mai, J.K., Paxinos, G., Voss, T., 1997. Atlas of the human brain, Vol., Academic Pr, San Diego.

Morel, A., 2007. Stereotactic atlas of the human thalamus and basal ganglia, Vol., Informa HealthCare USA, Inc., NewYork.

Mori, S., Tournier, J.D., 2014. Chapter 8 - Moving Beyond DTI: High Angular Resolution Diffusion Imaging (HARDI). Vol., ed.^eds. Academic Press, San Diego, pp. 65-78.

Pelzer, E.A., et al., 2013. Cerebellar networks with basal ganglia: feasibility for tracking cerebello-pallidal and subthalamo-cerebellar projections in the human brain. The European journal of neuroscience.

Perrin, M., et al., 2005. Fiber tracking in q-ball fields using regularized particle trajectories. Information processing in medical imaging : proceedings of the ... conference. 19**,** 52-63.

Reese, T.G., et al., 2003. Reduction of eddy-current-induced distortion in diffusion MRI using a twice-refocused spin echo. Magnetic resonance in medicine : official journal of the Society of Magnetic Resonance in Medicine / Society of Magnetic Resonance in Medicine. 49**,** 177-182.

Schaltenbrand, G., Wahren, W., 1977. Atlas for Stereotaxy of the Human Brain. In: Thieme. Vol. 2, ed.^eds., Stuttgart, pp. 84.

Wiegell, M.R., et al., 2003. Automatic segmentation of thalamic nuclei from diffusion tensor magnetic resonance imaging. Neuroimage. 19**,** 391-401.

**Figures**


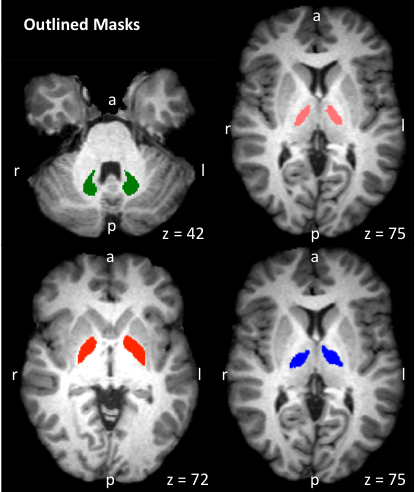


Figure 1

**Overview of masks derived from manual and atlas-based segmentation.**

Left: Outlining of the dentate nucleus (top; green) at axial slice z=42 and the pallidum (bottom; red) per manual segmentation at axial slice=72.

Right: Outlining of the ventrolateral thalamic nucleus per manual segmentation (top; pink) and atlas-based segmentation (bottom; blue) at the axial slice z=75.
